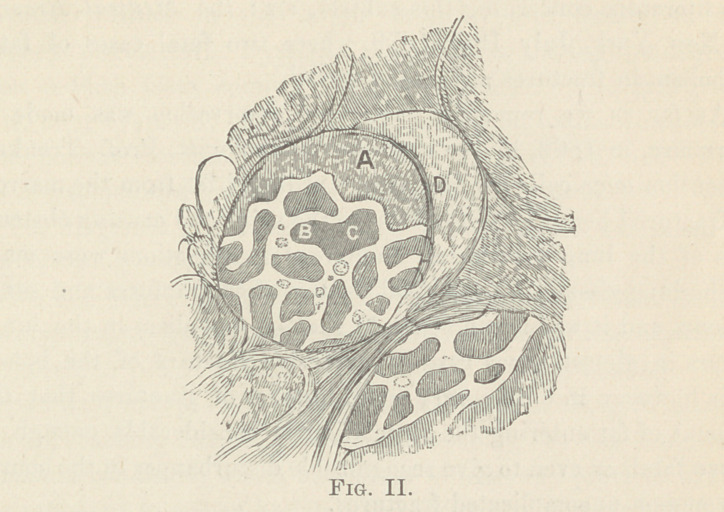# Diffuse Multiple Capillary Fat Embolism of the Lungs and Brain as a Fatal Complication in Common Fractures; Illustrated by a Case

**Published:** 1879-12

**Authors:** Chr. Fenger, J. H. Salisbury


### ARTICLE III.

DIFFUSE MULTIPLE CAPILLARY FAT EMBOLISM OF THE LUNGS AND BRAIN AS A FATAL COMPLICATION IN COMMON FRACTURES, ILLUSTRATED BY A CASE. By DR. CHR. FENGER and DR. J. H. SALISBURY. A paper read before the Chicago Medical Society, Nov. 17th, 1879.

In calling your attention to the above-named serious but very rare disease, we shall first quote the history and post-mortem examination of a case observed last summer in Dr. E. W. Lee's surgical ward in Cook County Hospital, and afterwards make some general remarks on the main features of the subject.

*History.*—The patient, Mrs. B., a housewife aged 45, and a native of Ireland, was admitted to Cook County Hospital, July 25th, 1879.

On admission, the patient stated that she had fallen from the roof of a kitchen to the ground, a distance of 3 meters, striking upon her left side. On examination, evidences of a fracture of the upper part of the shaft of the left femur were found.

The leg was placed in a comfortable position, but no permanent dressing was applied. Morphia was given *pro re nata.*

July 26th.—The patient was very restless, but did not complain of much pain.

July 27th.—In the morning the patient seemed to be sleeping quietly, but the respirations were quite rapid; 1 p.m., the patient was still unconscious; she could be roused somewhat, but did not become conscious; the pupils responded to light; 5 p.m., she had some slight spasms; the jaws were firmly set for a few minutes; 7 p.m., pulse 112, somewhat weak; temperature 38.5 ($101\frac{1}{4}$ F.); respirations, 40 per minute, regular. The patient was still comatose, face pale, lips slightly bluish. The movements of the thorax were natural. Upon percussion, the dullness of heart, liver and spleen was found to be within the regular boundaries. Auscultation showed the sounds of the heart to be normal. Over the lungs the normal vesicular breathing was heard. No râles were heard, either with inspiration or expiration. The posterior parts of the lungs were also natural. The abdomen was natural. The pupils responded to light, and were equal in size. There was no local paralysis in any part of the body. The urine contained no albumen.

July 28th.—The symptoms were about the same, except that all over the lungs were heard the coarse râles which usually occur in the agony.

Dr. Fenger saw her, and made the diagnosis of diffuse multiple capillary fat embolism of the lungs. Prognosis, fatal.

She died in the afternoon of July 28th.

Dr. Salisbury noticed about the patient an indescribable sweetish odor.

*Autopsy.*—To the coroner, General Mann, we owe our thanks for his kindness, which enabled us to hold the exceedingly interesting post-mortem. There were present General Mann, Drs. McWilliams, Merriman and Lee, of the hospital staff, besides the internes of the hospital.

The autopsy was made twenty hours after death. The rigor mortis was well marked. The subcutaneous adipose tissue was abundant. The striated muscles appeared natural. In the pericardium was found about 15 cubic centimeters of thin yellow fluid. The heart was natural in shape and size, but flabby. The valves and endocardium were natural. The heart muscle was somewhat grayish. The heart and large vessels contained dark fluid blood, as in strangulation. Small drops of fat were found swimming on the blood. Some old adhesions existed in the left pleural cavity. Nothing abnormal was found in the pleura costalis, nor in the pleura pulmonalis. In the subpleural tissue were many small ecchymoses, up to the size of a pin's head.

*Left Lung.*—The surface of the whole lung had a peculiar red, spotted appearance, which was most marked in the anterior parts of the lobes. The cut surface of the lung presented the same appearance. Some parts were quite white, which was due partly to anæmia, but chiefly to emphysema along the anterior borders.

The posterior part of both lungs was congested and somewhat œdematous. There was no capillary bronchitis. The bronchial mucous membrane was somewhat injected, but there were no ecchymoses and no mucus except in the largest tubes.

In one place, at the base of the lower lobe of the right lung, were some larger ecchymoses. One was as large as a lobule, M. .008 in diameter. These ecchymoses were mostly subpleural. The cranium was rather thick, but otherwise natural. The dura mater was natural. The lateral ventricles contained a little clear serous fluid. The brain tissue of the hemispheres was natural, and not particularly anæmic. On the cut surface of the hemispheres, especially in the white substance, were found numerous ecchymoses, appearing as small, round, dark, blood-red points, varying in size from points scarcely visible up to 1 millimeter in diameter. These were found all through the white substance, and a few were found in the gray. The same spots were found in the cerebellum, and a group of them in the anterior part of the pons varolii, and some in the corpus callosum. The vessels at the base of the brain were natural. The substance of the large ganglions was natural.

No fluid was found in the abdominal cavity. The peritoneum was natural. The spleen was of natural shape and size, but on the surface were seen several small, dark irregularly shaped spots 4 millimeters in diameter, which seemed to be superficial hæmorrhages.

The liver was grayish and anæmic, but there were no ecchymoses. The kidney was of natural shape and size, flabby, but otherwise normal. The uterus and bladder were normal. In the fundus of the stomach were small ecchymoses in a limited space of 2.5 Cm. in diameter. Otherwise the mucous membrane of the intestines was normal. In the upper part of the left femur between the 1st. and 2nd. third, was a complete transverse fracture, surrounded by the usual amount of coagulated blood, filling the surrounding inter-muscular spaces. The substance of the fractured bone was normal. The marrow in the canal of the shaft was yellow from infiltration with fat, as we usually find it in elderly persons. No traces of inflammation were seen in or around the fracture. There were no coagula in the larger of the surrounding veins. The femoral vein contained dark fluid blood with no visible fat drops in it.

Microscopical examination showed the following interesting features:

Small pieces cut off with the scissors from the surface of the lung showed the smaller arteries and some of the capillaries of the pleural tissue as a whitish-yellow refracting net-work, owing to the injection and filling up of those vessels with liquid fat. Sections from the interior of the lung tissue showed a fine, more or less complete injection of liquid fat, in the net-work of capillaries surrounding and protruding into the air cells.

The following sketches will show the above mentioned features:

FIG. I.

Figure I, shows one complete air cell, *a*, and three incomplete ones, *b*, *c*, *d*. In the interstitial tissue, between these four air cells, you see besides the usual characteristic elastic fibers, a light net-work of injected capillaries, a loop of which protrudes at *e* and *f* in high relief from the internal wall of the air cell; at *g*, part of the net-work of capillaries at the bottom of the air cell is visible, on account of the injection with fat.

Fig. II shows an almost complete injection of the capillary net-work at the bottom of the air-cell *a*. In the interior of this net-work we find the homogeneous aspect of the fat, interrupted by small, round, granular bodies of the size of a white blood corpuscle. The above mentioned small bodies may be either white blood corpuscles still clinging to the wall of the capillary afte the oily fat has set in, or leucocytes brought up with the fat from the marrow where they are usually found in great numbers.

The specimens we hereby show in the microscope do not exhibit the above described condition so distinctly now, because the fat,

FIG. II.

in the course of the elapsed three months has, in most places, run out from the cut branches of the capillaries and shows itself now as round, refracting, fat drops scattered all over in the tissues. Still, the capillary injection is visible in some places and the filling up of the smaller arteries with fat can be easily enough recognized—as shown in the microscope with the low power. In another microscope we show a section of the lung tissue with empty capillaries only to call attention to the fact that here the capillaries are not visible at all, and, unless the capillaries are filled with blood or some colored artificial injecting fluid, we will not be able to see a trace of them. The microscopical examination of the small hæmorrhages in the brain tissue, showed the sub-capillary arteries in the center of the hæmorrhage filled with fat in the same manner as in the lungs. In none of the other organs of the body were we able to discover any fat in the smaller vessels.

Upon adding osmic acid in the sections of lung tissue, the fat in the vessels is colored black and the vessels then appear as if they were filled with some black injecting fluid. This renders the demonstration of the presence of fat much easier than by the examination of unstained specimens.

We are indebted to the kindness of Dr. Merriman for part of the literature concerning this subject, viz: the *Medical Record*, of New York, July 19th, 1879, where two fatal cases of fatty embolism in fractures are briefly stated.

As far as we remember, the first observation was made in Germany, in 1862, by the renowned pathologist, Prof. Trenker. Attention once called to the danger of liquid fat from the marrow of fractured bones gaining access to the veins and causing obstruc tion of the lung capillaries, numerous examinations were made of the lung tissue, in occasional deaths after fractures and other lesions, and it was found (Orth *) that fat embolism in the lungs occurs in almost every case of extensive fracture of the bones. It is however in only a very small number of fractures that the amount of fat entering the circulation is considerable enough to prove fatal, or even to give recognizable disturbances in the course of common uncomplicated fractures.

Further investigations by Flournoy and V. Recklinghausen in the necropsy theater at Strasbourg, showed that slight diffuse fatty embolism could be found in 10 per cent. of a series of 260 dead bodies. Up to 1879, Egli Sinclair † had gathered records of 140 reported cases, and he found the etiology to be limited to one of the following three morbid conditions: 1. Extensive contusion or laceration of soft parts, containing abundance of adipose tissue. 2. Fracture, with extensive lesion of the marrow of the bones, and, 3. Osteomyelitis—chronic as well as acute inflammation of the marrow of the bones.

The most severe cases of fatty embolism however set in after fractures; *e. g.* In 140 cases, death ensued in 18; that is, 13 per cent. Of these 18 deaths, 16 occurred in the 84 cases of fracture.

*Symptoms and Diagnosis.*—The symptoms, as Egli Sinclair gives them, from cases of fatty embolism in extensive fractures,

---

* Orth, Diagnosis in Pathological Anatomy. Translated by Shattuck. New York, 1878. P. 160.

† Egli Sinclair, Ueber Fettembolie. Correspondenzblatt fur Schweizer Aerzte V. 6, 1879. St. Petersburg *Med. Wochenschrift*, N. 23, 1879; *Allgemeine Medicinisch Central-Zeitung*, Berlin, 2 Juli, 1879, p. 683.

are as follows: Unexpected, rapidly increasing, general debility; then the symptoms from insufficience or entire absence of oxidation of the blood; respirations from 40 to 60 in the minute; pulse weak and frequent; temperature often somewhat augmented. Râles in the larger bronchi, and finally in the trachea (præmortal). Dyspnœa sometimes to the highest degree; then reddish foam coming out of the mouth. The face grows pale, later cyanotic; the extremities get cold, pupils contracted. The patient becomes somnolent, finally comatose, and death ensues, sometimes preceded by vomiting and spasms.

The diagnosis in the case which we have related was based on the following reasoning:

We had before us a previously healthy person with a simple, uncomplicated fracture of the femur, that from the beginning promised to run the usual benign course towards healing. The second day, except some restlesness, there was nothing to indicate the approaching danger. The third morning she was found in a comatose condition, which had set in without any previous suffering sufficient to waken her from her sleep, which means that the grave symptoms, as usual in these cases, set in suddenly. Besides this comatose condition, we find no fever of any account. A temperature of 101¼° is the usual aseptic and innocent rise in temperature that will be found (R. Volkman, in 11 out of 14 *) in most of the fractures of the femur not treated with immovable dressings. The physical examination does not show any morbid symptom in the organs of the thorax and the abdomen. The urine shows that there is no disease of the kidneys and no diabetes. As to the brain, we find no symptoms of a local disease. There is no paralysis, equal pupils and no symptoms of pressure, such as stertorous breathing, etc.

The only positive symptoms able to lead attention in the direction of the seat of serious trouble, were the cyanosis, paleness of the face, bluish hue of the lips and the augmented number of respirations—40. These symptoms evidently pointed to the lungs. As now the air-cells as well as the bronchi were normal, we must place the trouble in the circulatory system of the lungs, thrombosis or embolism in a great part of the pulmonary vessels.

* I. Volkman, Samlung Klinischer Vortrage, p. 1023. Ueber Septisches und Eseptisches Wundfieber.

A spontaneous thrombosis in the trunk and branches of the pulmonary artery can take place in endarteritis of this artery. But this disease is as seldom found here as endocarditis in the right heart. Embolism could occur from a loosened thrombus in any part of the venous system from the right ventricle or auricle but here was no previous heart disease and no previous exhausting febrile disease.

The only peripheral diseased place to be found was this recently fractured femur. Around a fracture, thrombosis in the larger veins is not uncommonly found (F. Durodié *). The thrombi from the smaller veins formed round every fracture extending out into larger and larger veins, causing probably part of the œdema accompanying so many fractures of the extremeties. Loosening of part of these thrombi and subsequent embolism of the lungs is rare but takes place in one case out of three hundred (Durodié). A sufficiently large aseptic embolus in both of the main branches of the pulmonary artery might give a similar series of symptoms ending in death. But the formation of these peripheral venous thrombi and their subsequent detachment and entrance into the circulation take a much longer time than 48 hours, and consequently we were obliged to abandon this explanation of the symptoms. Finally there was left no other diagnosis that would correspond to the symptoms of the case than the fatty embolism of the lung capillaries, i. e. the introduction into the circulation of liquid fat in sufficient quantity to make the greater part of the lung capillaries impassible for the blood. The moderate acceleration of the pulse and the not extreme cyanosis are easily explained by the difficult passage of the blood through the lungs from the venous system over-filled with blood. The weakness of the radial pulsation is a natural consequence of the diminished quantity of blood in the arterial system. The comatose condition may be explained by the want of blood supply to the brain and the medulla oblongata (Wagner †) probably combined with accumulation of carbonic acid in the blood. Whether

* F. Durodié Etude sur les thromboses et l'embolie veineuses dans les contusions et les fractures. Thèse, Paris, 1874.

† Wagner, Manual of General Pathologie. Translated by Van Duin; New York; 1876; p. 209.

the multiple capillary embolism in the brain in our case contributed to the depression of the cerebral functions or not, cannot be decided.

In a number of the reported cases of this kind the fatty embolism has caused sudden death. (Wagner, loc. cit).

In one of Déjerine's cases death occurred in two and one-half hours, in the other, thirty-six hours after the fracture was received. The report of his cases does not give any information about the duration of the grave symptoms. About this we cannot tell anything for want of the original reports of previous cases. Our case, though fatal, did not take a very rapid course, which was so far interesting, as it gave sufficient time (the grave symptoms lasted over thirty-six hours) to have the diagnosis based upon a minute examination of the symptoms.

*Prognosis.* — The prognosis depends upon the quantity of the circulating fat, and upon the strength of the heart's action. If the right ventricle can get and keep up power enough to push the fat through the lungs, then the immediate danger will be overcome. An extensive fracture, as the source of the embolism, will make the prognosis worse 20 to 40 per cent. than lacerated soft tissues or osteomyelitis.

*Treatment.* — The natural treatment will be to stimulate the action of the heart in the hope that an increased *vis a tergo* can drive part of the fat through the lung capillaries, out into the aortic system (digitalis, alcoholics, etc). When the immediate danger from the pulmonary system can be overcome, then the organism will gain time to get rid of the fat, presumably by transforming it into soluble soaps through the action of the alkalies in the blood. Merely hypothetically, we should advise to keep the fractured bone or the diseased part scrupulously immovable, with the view of preventing any more liquid fat from escaping from the tissues. As to this point, we must remember that in the marrow as well as in the adipose tissue, the fat is contained in so-called fat-cells, i. e. membranous sacs. These membranes must be destroyed or torn open before their contents of liquid fat can gather in a fluid, movable mass; and it is in this condition of the fat that the danger lies, as we do not find the fat-cells or sacs but only their contents in the capillaries of the lungs.

## Figures and Tables

**Fig. I. f1:**
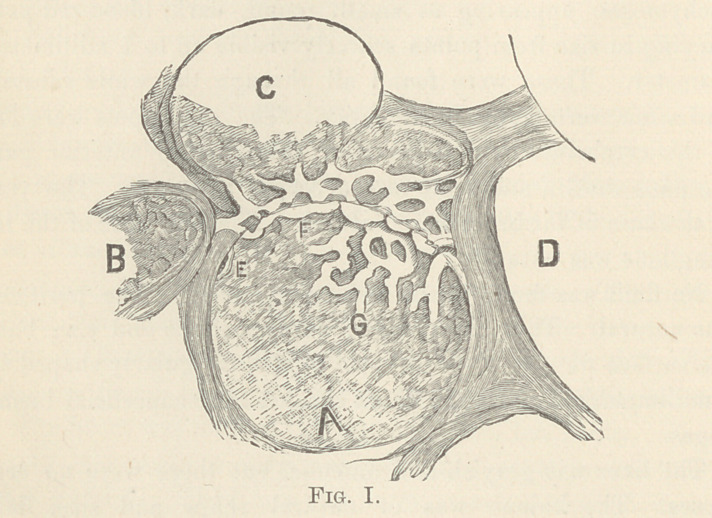


**Fig. II. f2:**